# Dual pH- and temperature-responsive poly(dimethylaminoethyl methacrylate)-coated mesoporous silica nanoparticles as a smart drug delivery system

**DOI:** 10.1038/s41598-023-47026-7

**Published:** 2023-11-18

**Authors:** Sina Ramezanian, Jafarsadegh Moghaddas, Hossein Roghani-Mamaqani, Azim Rezamand

**Affiliations:** 1 Chemical Engineering Faculty, Sahand University of Technology, P.O. Box 51335-1996, Tabriz, Iran; 2 Transport Phenomena Research Center, Chemical Engineering Faculty, Sahand University of Technology, P.O. Box 51335/1996, Tabriz, Iran; 3 Faculty of Polymer Engineering, Sahand University of Technology, P.O. Box 51335-1996, Tabriz, Iran; 4 Institute of Polymeric Materials, Sahand University of Technology, P.O. Box 51335-1996, Tabriz, Iran; 5 Pediatric Health Research Center, Tabriz University of Medical Sciences, Tabriz, Iran; 6 Department of Pediatrics, Faculty of Medicine, Tabriz University of Medical Sciences, Tabriz, Iran

**Keywords:** Drug delivery, Polymers

## Abstract

A robust drug delivery system was created by grafting poly(dimethylaminoethyl methacrylate) (PDMAEMA) onto silica nanoparticles with two different lengths using an in situ atom transfer radical polymerization, resulting in the formation of a pH- and temperature-sensitive shell. The high molecular weight PDMAEMA demonstrated effective controlled drug release, and prevented drug release in healthy cells. Drug release occurred through polymer shell protonation at pH 5. The critical temperature of 41 °C facilitated rapid solvation of the shell polymers in the blood, preventing tissue accumulation and reducing toxicity compared to systems with lower critical solution temperatures. Field-emission scanning electron microscopy analysis and nitrogen adsorption/desorption analysis showed that the nanoparticles have a fine network, mesoporous structure, and a mean size of around 17 nm that show their excellent capacity for loading drugs. Fourier-transform infrared spectroscopy showed that all the modification steps and polymerization were successfully implemented. Thermogravimetric analysis showed PDMAEMA chains with two different lengths grafted onto the nanoparticles. Transmission electron microscopy analysis also showed grafted polymer chains on the hybrid nanoparticles. The release profile of model cancer drugs (doxorubicin and methotrexate) varied with pH and temperature, with high molecular weight PDMAEMA shells effectively preventing drug release at neutral pH. In vitro analysis using the HeLa cell line showed minimal toxicity in blank samples and significant release profile in acidic environment.

## Introduction

Cancer is an uncontrollable grow of cells in specific part of the body and their spread to another area. Annually cancer accounts for a quarter of the deaths in the United States^1, 2^. Cancerous environment is different from healthy tissue^3, 4^. Understanding the disparities between healthy and cancerous tissues, such as lymphatic system disorders^5, 6^, angiogenesis^7, 8^, and vascular problems^9, 10^ is crucial for the development of targeted treatment techniques. Tumors exhibit permeability and retention, allowing the accumulation of nanoparticles smaller than 400 nm due to their rapid growth and formation of new blood vessels^11, 12^. Consequently, nanoparticles below this size threshold can exit the bloodstream and accumulate within the tumor tissue^13, 14^.

Chemotherapy is one of the primary methods of cancer treatment^15^; however, its effectiveness is limited due to side effects and immune system filtration^16^. Common side effects of chemotherapy include Alopecia, bone marrow suppression, stomach erosion, and cardiomyopathy^17^. Consequently, there is a need to develop new treatments that selectively target tumors^18^. The advancement of nanotechnology has led to the development of nanoparticles with targeting capabilities^19^. These nanoparticles accumulate in tumor tissue to reduce the side effects of chemotherapy^20^. The use of nanoparticles in chemotherapy offers several benefits, including preventing from drug destruction in the body, increasing drug delivery to the tumor, precise tumor targeting, and controllable treatment stages^21^. The advantages of drug delivery systems have prompted extensive research to develop optimal systems with high efficiency and minimal toxicity^18, 22^.

Mesoporous silica nanoparticles (MSNs) have emerged as a promising option for tumor treatment^23, 24^. Their unique properties, including porous structure, high hydrophilicity, high surface-to-volume ratio, surface modification ability, and diverse dimensions, shapes, and structures, make them suitable candidates for cancer therapy^25–28^. However, using these particles alone without any modification is insufficient for an effective drug delivery system^29^. Therefore, drug carriers are modified to be responsive to various stimuli, such as temperature, enzymes, electromagnetic fields, infrared radiation, redox reactions, and ultrasound, to prevent drug release in healthy tissue and the circulatory system^30^. In addition, the tubular pores of MSNs can be blocked using stimuli-responsive materials^31^. Controlling drug release in MSNs can be achieved through three methods of surface coating of nanoparticles^32^, using caps for pores^33^, and drug attachment to the inner wall of the nanoparticles^34^.

One of the most commonly used responsive materials to render nanoparticles sensitive is pH- and temperature-responsive materials. This is particularly relevant for tumor treatment, as the pH of tumors is lower than that of healthy tissue due to the high lactic acid production^35^. Furthermore, the intracellular environment of tumors exhibits a lower pH range of about 4.5–5.5. The pH difference between tumor and healthy tissue allows for the release of drugs specifically at the tumor site^36^. Additionally, the high metabolic activity of tumor cells leads to local temperature elevation compared to normal tissues^37, 38^. Taking advantage of these differences, stimuli-responsive silica nanoparticles are employed in this work to precisely control the release of cancer drugs. Atom transfer radical polymerization (ATRP) is a highly applicable type of controlled radical polymerization systems^39–41^. This method has been commonly employed for synthesis of smart polymers with controlled molecular weight and structure^42, 43^. Temperature and pH stimuli-responsive materials have found wide applications in biomedicine and biotechnology^44–46^. Poly(dimethylaminoethyl methacrylate) (PDMAEMA), for instance, exhibits dual sensitivity to temperature and pH due to its aliphatic tertiary amino groups^47^. The pK_a_ of PDMAEMA is about 7.0^48, 49^, and its lower critical solution temperature (LCST) of around 32–53 °C depends on pH of media, molecular weight of the polymer, and salt concentration.

Today, numerous smart systems have been developed with applications as drug delivery systems. The smart control and targeted release of drugs into cancerous tissue present crucial challenges in this field, aiming to reduce the side effects of chemotherapy. To address these challenges, the distinctions between healthy and cancer cells, such as pH variations, temperature differences, and specific enzymes present in tumors, are utilized to enhance the intelligence of nanoparticles and enable drug release exclusively within the tumor. In this study, a pH- and temperature-sensitive polymer was grafted onto MSNs to regulate the release of anticancer drugs. Subsequently, the study examined various drug loading and release characteristics of the PDMAEMA-coated nanoparticles, considering different lengths of PDMAEMA chains.

## Experimental

### Materials

Cetyltrimethylammonium bromide (CTAB, Merck Millipore, 98%) as the surfactant, tetraethoxysilane (TEOS, Merck Millipore, 99%) as the silica reagent, ethanol (C_2_H_5_OH, Merck Millipore, 99%) and tetrahydrofuran (THF, Merck Millipore, 99%) as the solvents, and 3-triethoxysilylpropylamin (APTES, Merck Millipore, 98%) as the surface modifier of the MSNs, and 2-bromoisobutyryl bromide (Merck Millipore, 98%) as the ATRP initiator were utilized. Triethylamine (TEA, Merck Millipore, 99%) was refluxed with 4-toluenesulfonyl chloride (tosyl chloride, Merck Millipore, 98%) to eliminate the primary and secondary amines. *N*,*N*,*N*′,*N*″,*N*″-Pentamethyldiethylenetriamine (PMDETA, Merck Millipore, 99%) was mixed with CaH_2_ overnight and distilled under vacuum before usage to remove the impurities. Dimethylaminoethyl methacrylate (DMAEMA, Merck Millipore, 99%) was passed through an alumina column to eliminate the inhibitors. CuBr (Sinopharm Chemical Reagent Co. 98%) was utilized as a polymerization catalyst. Dialysis tubing cellulose membrane (12,000 KDa, Sigma-Aldrich) was prepared for releasing cancer drugs. Doxorubicin (DOX) and methotrexate (MTX) as the model drugs were purchased from EBEWE Pharma. Methanol, toluene, acetone, and all the other material were used as received.

### Preparation of the drug carriers

#### Synthesis of MCM-41

MCM-41 was prepared by a hydrothermal method. In this regard, CTAB and TEOS were dissolved in deionized water, and NaOH solution (2 molar) was added dropwise to the solution up to pH 10.5. In the aging step, the mixture was mixed for 2 h at ambient temperature, and then the solution was moved to a stainless-steel autoclave reactor and transferred to an oven at 140 °C for 72 h. Then, the product was washed with deionized water, filtered, and dried at 110 °C for 6 h. Finally, the white powder was calcined for 6 h at 600 °C in a furnace.

#### Modification of MCM-41 with amine groups to prepare MSNs-NH_2_

2.5 g of MSNs was dispersed in 75 mL of ethanol for 30 min. Then, 20 mL of triethylamine was added and mixed for 2 h at 40 °C. After that, 1.5 mL of APTES dissolved in 20 mL ethanol was slowly added to the mixture and stirred for 12 h at 40 °C. Then, the resulting material was centrifuged at 8000 rpm for 10 min. Finally, the particles were washed three times with 80 mL of acetone and dried at 60 °C in vacuum oven to obtain MSNs-NH_2_.

#### Modification of MSNs-NH_2_ with ATRP initiator to prepare MSNs-Br

0.5 g of the modified nanoparticles were dispersed in 40 mL of THF for 30 min to obtain a homogeneous suspension. Then, 38 mL of triethylamine was added to the suspension. After that, 1.3 mL of 2-bromoisobutyryl bromide and 30 mL of THF were added to the mixture dropwise and mixed at 0 °C for one hour. Then, the solution was kept at ambient temperature for 36 h. The suspension was washed twice with THF, then with deionized water and ethanol at a volume ratio of 1/1 for three times. The suspension was passed through a 2 μm PTFE filter and dehydrated in a vacuum oven at 60 °C to yield MSNs-Br.

#### Synthesis of PDMAEMA-coated MSNs (MSNs-*g*-PDMAEMA)

Firstly, 0.1 g of MCM-41 was dispersed in 0.5 mL of DMAEMA and 5 mL of THF for 10 min. After that, the solution was moved to a reactor including 4.5 mg of CuBr with an N_2_ atmosphere. Then, 6 µL PMDETA was added to the reactor and heated to 75 °C for 24 h. In calculations of molar ratios, CuBr: PMDETA: DMAEMA: nanoparticle ratio of [1]:[1]:[100]:[50] was used for the synthesis of the short chain PDMAEMA-modified nanoparticles (MSNs-*g*-PDMAEMA1) and the molar ratio of [1]:[1]:[500]:[50] was used for the synthesis of the large chain PDMAEMA-modified nanoparticles (MSNs-*g*-PDMAEMA5). These modification steps are shown in Fig. S1 (Supporting Information).

#### Separation of the CuBr catalyst from MSNs-*g*-PDMAEMA

CuBr is a toxic material and should be separated from the carrier. CuBr catalysts in MSNs-*g*-PDMAEMA cannot be removed by using an alumina column due to their trapping in the column. Therefore, the MSNs-*g*-PDMAEMA carrier was dispersed in ethanol/THF (10:1 v/v) for 1 h and centrifuged to collect the nanocarrier. To increase the purity of the carrier, the washing process was repeated several times with toluene.

### Characterization

The morphology and size of MCM-41 and MSNs-*g*-PDMAEMA5 were investigated by transmission electron microscopy (TEM, 200 kV, JEM-2100F). Fourier transform infrared (FT-IR) analysis was used to confirm the modification of MCM-41 with amine and bromine groups and also and the polymer chains. FT-IR measurements were performed by Tensor 27 spectrometer (Bruker, Germany) in the wavenumber range of 400–4000 cm^−1^. For this purpose, a KBr disk was used to make the samples powder transparent to infrared waves. Thermal gravimetric analysis (TGA) was performed with a Q600 (TA, USA) under a nitrogen flow in the temperature range of 50 to 800 °C with a 10 °C/min heating rateNitrogen adsorption/desorption isotherms were recorded on a BELSORP MINI II (BEL Engineering, Italy). Lower critical solution temperature (LCST) of MSNs-*g*-PDMAEMA5 was observed by UV–vis spectroscopy M51 (BEL Engineering, Italy) by increasing temperature with a rate of 1 °C/min. Field-emission scanning electron microscopy (FE-SEM) was utilized to investigate the morphology and size of the MCM-41 clusters by MIRA3 (TESCAN, Czech Republic). For this purpose, MSNs were coated with Au. Zeta potential of MCM-41 and MSNs-g-PDMAEMA5 were obtained in deionized water by Nanotrac Wave (Microtrac, USA).

#### Loading of MTX and DOX drugs

To load the drug molecules on MSNs-*g*-PDMAEMA, DOX and MTX drugs were dissolved in deionized water (0.2 mL of each drugs/2 mL of deionized water). Then 0.01 g of the PDMAEMA-coated nanoparticles was added to a solution and stirred in the dark for 24 h. Then, carriers were washed three times with deionized water and separated by centrifugation, and their effluent was used to ascertain the amount of drug loading.

The following equations were used to calculate the amount of drug loaded:

1 $${\text{Loading}}\,{\text{content}}\,\left( {{\text{wt}}\% } \right) = \left( {{\text{Weight}}\,{\text{of}}\,{\text{drug}}\,{\text{in}}\,{\text{carrier}}/{\text{Weight}}\,{\text{of}}\,{\text{nanoparticles}}} \right) \times {1}00$$

2 $${\text{Entrapment}}\,{\text{efficiency}}\,\left( {{\text{wt}}\% } \right) = \left( {{\text{Weight}}\,{\text{of}}\,{\text{drug}}\,{\text{in}}\,{\text{carrier}}/{\text{Initial}}\,{\text{weight}}\,{\text{of}}\,{\text{drug}}} \right) \times {1}00$$

#### Release of DOX and MTX drugs

10 mg MSNs-*g*-PDMAEMA1 and MSNs-*g*-PDMAEMA5 loaded with DOX and MTX were dispersed in 1 mL of PBS (0.01 molar, pH 7.4 and pH 5), and then moved into the dialysis bags (cut-off 12,000 KDa). Afterward, the bags were immersed in PBS (22.4 mL) and incubated at different temperatures (25, 37, and 41 °C). For each UV–Vis test at a specific time, 3 mL of PBS was taken out and analyzed. The concentration of DOX and MTX was ascertained by comparing the absorbance at 478 and 373 nm with the standard curve.

#### Cell viability analysis

The cytotoxicity of nanoparticle was assessed using the 3-(4, 5-dimethylthiazole-2-yl)-2,5-diphenyl tetrazolium bromide (MTT) assay. HeLa cell lines have been seeded in 96-well plates at a density of 5000 cells/well. After 24 h, the cells were treated with various concentrations of MSNs-*g*-PDMAEMA, MSNs-*g*-PDMAEMA-MTX, and MSNs-*g*-PDMAEMA-DOX and incubated at 37 °C. Fresh medium was replaced instead of transfection medium after 6 h, and the plates were incubated again for 24, 48, and 72 h. After removing the medium, the cells were washed with PBS, and 50 μl of MTT solution (1 mg/ml) was added to plates and incubated in the dark. After 4 h, the medium was eliminated, and DMSO (200 μl) was added to the plates. For evaluation of plates, using a colorimetric experiment could measure the absorbance at 570 nm. Cell viability has been schemed by % viable cells (y-axis) against the concentration of carriers. Inhibitory concentration to kill 50% of the cell population (IC50) of MTX and DOX was the maximum dose for treating Hela cells.

## Results and discussions

### Characterizations of the neat and modified MCM-41 nanoparticles

**Figure 1:**
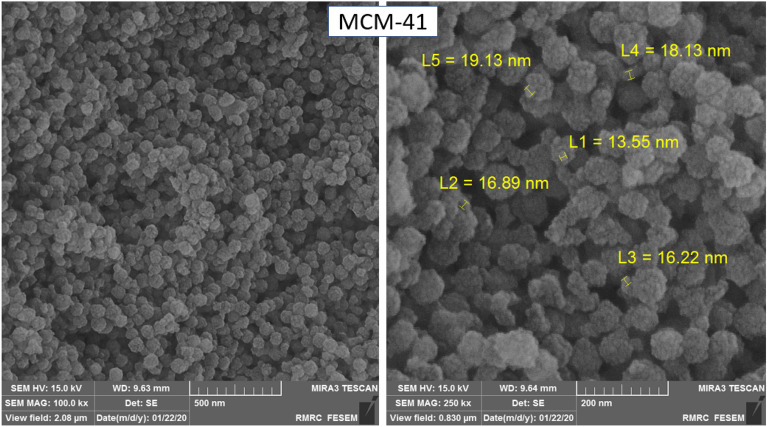
FE-SEM images of the MCM-41 nanoparticles.

After synthesis of MSNs (MCM-41), FE-SEM analysis was used to investigate its morphology and particle size. In these images, shape, size, and size distribution of nanoparticles were determined. These images are presented with two different magnifications in Fig. 1. According to Fig. 1a, the synthesized nanostructure clusters are separated, and the particles are slightly clumpy, which facilitates the dissolution of nanoparticles in the drug solution and better drug absorption. According to Fig. 1b, size of silica nanoparticles is about 15–20 nm. These images showed that the nanoparticles have spherical clusters. The modified nanoparticles also have a fine network and regular particles of about the same size.

**Figure 2:**
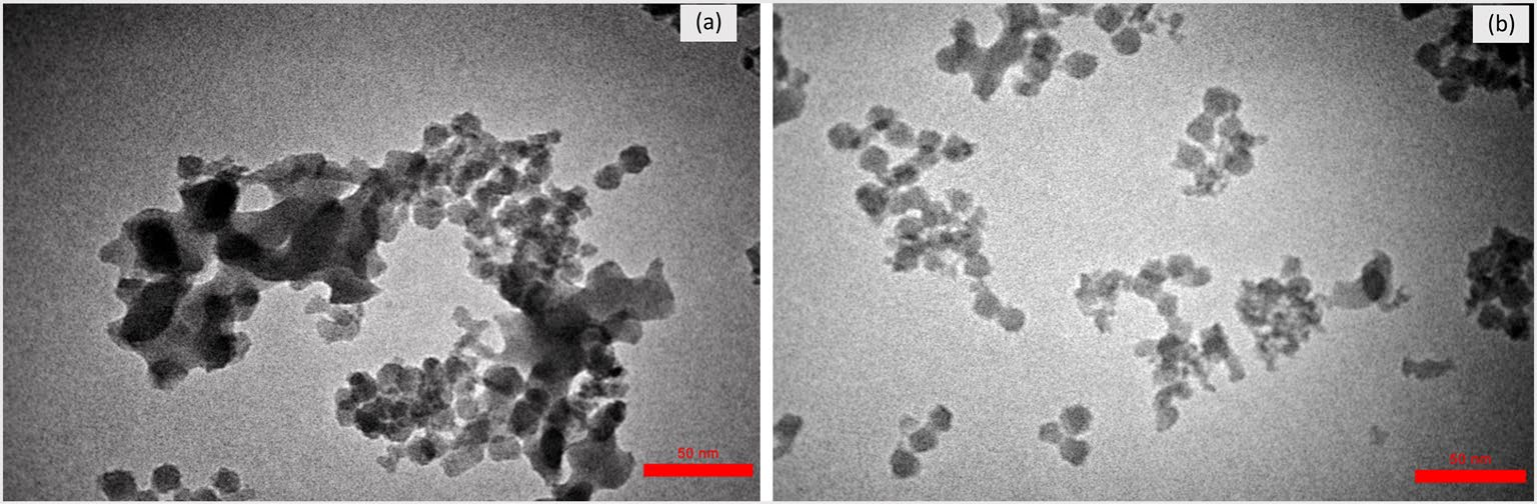
TEM images of (**a**) MCM-41 and (**b**) MSNs-*g*-PDMAEMA5.

Figure 2 shows the TEM images of MCM-41 (2a) and MSNs-*g*-PDMAEMA5 (2b). As shown in Fig. 2a, the synthesized MCM-41 presents a neatly round shape with prominent pore channels that are approximately 17 nm in size. Figure 2b shows that the nanoparticles coated with PDMAEMA chains have an almost spherical shape. MSNs-*g*-PDMAEMA5 nanoparticles were uniformly synthesized, and PDMAEMA chains were densely grafted on their surface. These images clearly showed mesoporous silica coated with a transparent layer of polymer and their typical core–shell structure. As a result, according to the EPR effect, nanoparticles of this size are inserted into the tumor easily. Also, the cell penetration size increases below 30 nm^11, 12^.

To ensure grafting of the surface modifiers and the polymer chains onto MSNs, FT-IR analysis was conducted, and the results are presented in Fig. S2 (Supporting Information), which was plotted using Origin Lab 2018 version. The red diagram (lowest diagram) is for MCM-41, where the peak at 3441, 1634, 961, and 1089 cm^−1^ is referred to as O–H, O–H–O, and Si–O bending vibrations and Si–O–Si stretching vibration, respectively. The blue diagram (second diagram from below) is for the MSNs-NH_2_, where the peaks at 2942 and 1530 cm^−1^ correspond to Si–OH and the amine groups, respectively. The yellow diagram (third diagram from below) is for MSNs-Br, in which the peaks at 1650 and 1540 cm^−1^ correspond to the bending vibration of C=O and N–H, respectively^50, 51^. The green diagram (highest diagram) is related to the MSNs-*g*-PDMAEMA5, in which the peaks at 1529, 1543, 1656, and 2950 cm^−1^ are respectively referred to as the bending vibration of methyl, ethyl, and C=O and C–H scratching vibration, which appeared in the FTIR curves after grafting PDMAEMA chains on MSNs^52–54^.

**Figure 3:**
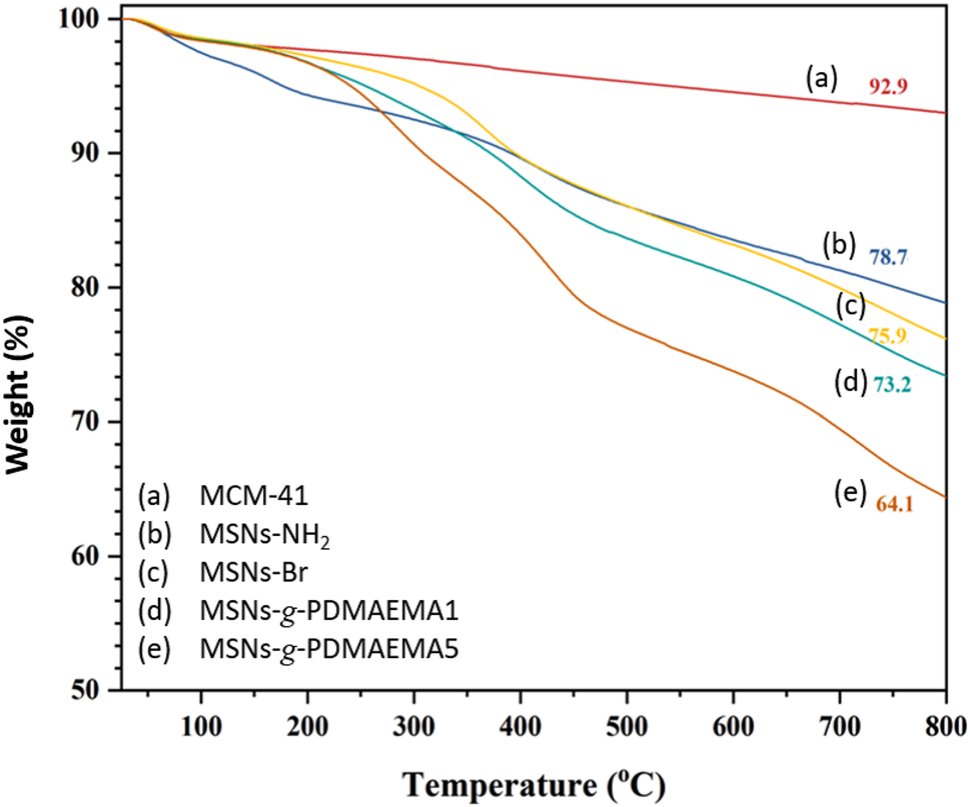
TGA results for the neat and functionalized MSNs.

TGA analysis was taken from the samples to observe the percentage of modification in each modification step and the content of grafted polymer chains with different lengths. In Fig. 3, the curve for the pure MCM-41 begins to lose weight at 100 °C, which is related to the moisture content of the MSNs at 100 °C. Weight loss of about 7.05% for the MCM-41 sample at 800 °C of it is because of complete moisture removal. At 100 °C, the MSNs-NH_2_ sample losses approximately 4% of its weight due to moisture, and degradation of amine functional groups started at about 200 °C. The sample weight reduction of about 22% at 800 °C indicates successful modification of the MCM-41nanoparticles with APTES. The sample modified with 2-bromoisobutyryl bromide started to lose 4% of its weight after 100 °C due to the humidity, and weight loss of about 24% till 800 °C can be assigned to the amine and bromine groups, where only 2% relates to the bromine groups degradation. Polymer degradation in the two PDMAEMA-coated MCM-41samples was started at 400 °C, where 2.7 and 11.76% weight loss was observed at 800 °C for the low and high molecular weight PDMAEMA-modified nanoparticles. This shows that the polymer chains were successfully grafted onto the nanoparticles with different lengths. DTG results are also shown in Fig. S3 (Supporting Information).

The charge of the cell membrane is negative, so the particle with a positive charge can penetrate to the cells. According to this fact, nanocarriers used for drug delivery systems should have a positive charge. MCM-41 nanoparticles have a negative charge (− 16 mV). After modification and grafting of polymer chains onto MCM-41 nanoparticles, zeta potential of the carriers changed to a positive charge (+ 34 mV), as shown in Fig. S4 (Supporting Information). Consequently, these carriers can easily penetrate into the cells.

**Figure 4:**
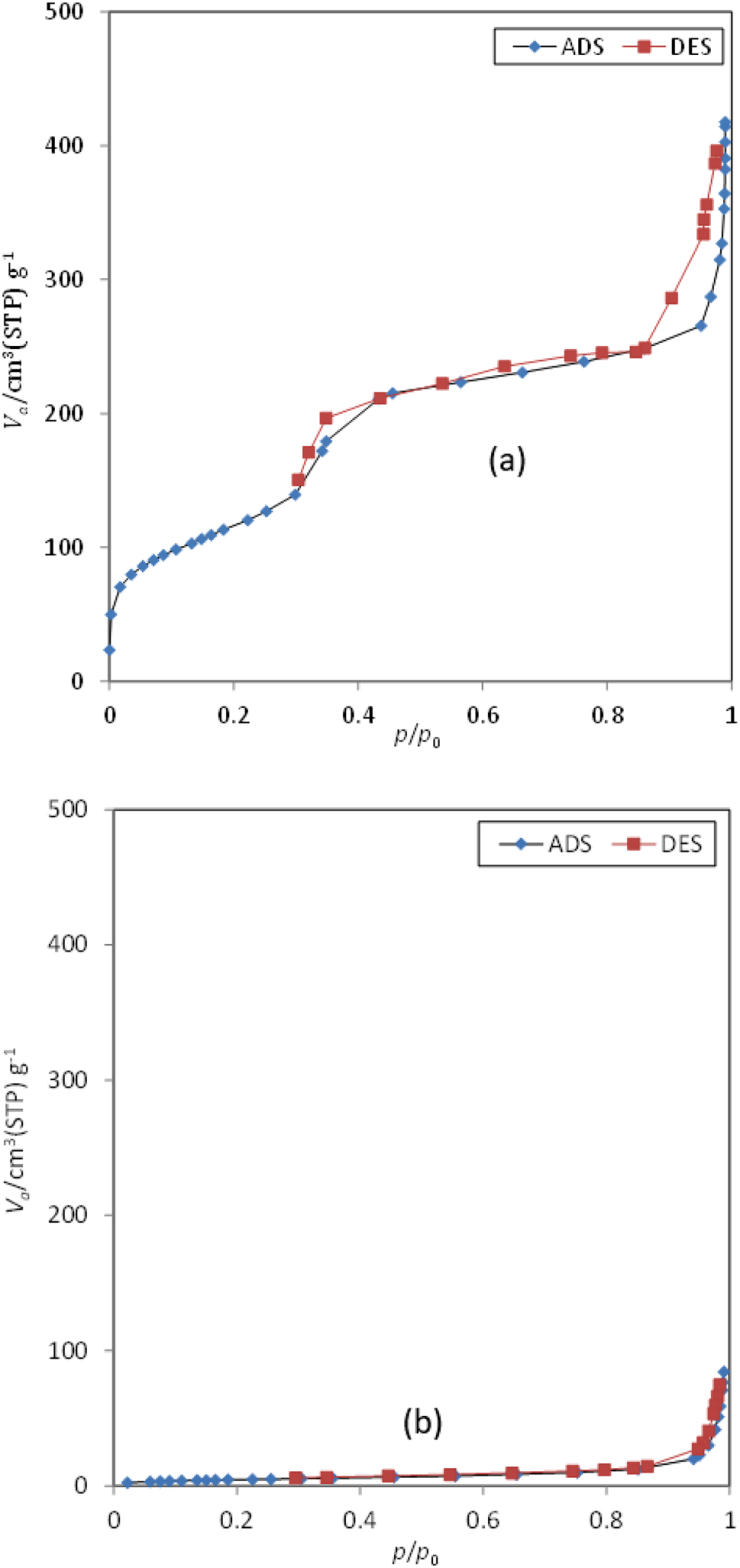
Adsorption/desorption isotherms for (**a**) MCM-41 and (**b**) MSNs-*g*-PDMAEMA5.

To investigate the surface properties and cavities of the prepared MCM-41 and MSNs-*g*-PDMAEMA5, BET-BJH analysis was taken from the samples, and the results are presented in Fig. 4. Accordingly, the diameter of the cavities is in the range that is not a limiting factor for penetration. On the other hand, because the volume of cavities is in the meso range, the drug adsorption operation is performed acceptably. The diagram of nitrogen uptake and desorption of the samples is shown in Fig. 4.

According to the type of hysteresis, the cavities are in the range of meso and macro, and the volume of cavities of the whole material is 0.65 at p_0_/p = 0.99. As shown in Fig. S5 (Supporting Information), the pores are is in the range of meso and macro. Surface properties of MSNs and MSNs-*g*-PDMAEMA5 are also added in Table 1.

**Table 1:** Surface properties of MSNs and MSNs-*g*-PDMAEMA5.

Sample	Mean pore diameter (nm)	Total pore volume (cm^3^/g)	Specific surface area (m^2^/g)
MCM-41	6.24	0.65	414.39
MSNs-*g*-PDMAEMA5	29.16	0.12	17.09

To find cloud points and plot the UCST diagram, the UCST thermoresponsive behaviour of the carrier was investigated by turbidimetry in water. For this purpose, nanoparticles were dispersed in water with different concentrations, and these mixtures were heated and cooled between 25 and 60 °C. The cooling-heating rate was 1 °C/min. Cloud point temperatures (TCPs) were ascertained at 50% of light transmittance at wavelengths of 600 nm throughout the cooling of solutions. The UCST point was around 41 °C.

### Drug loading on PDMAEMA-modified MSNs

To load the DOX molecules to the MSNs-*g*-PDMAEMA, 0.01 g of the nanoparticles with different chain lengths was added to a solution containing 0.4 mg of DOX and 2 mL of water. The mixture was stirred in the dark for 24 h. Similarly, for loading MTX, the nanoparticles were placed in a solution comprising 0.5 mg of MTX and 2 mL of deionized water. After 24 h, the carriers were separated from the drug solution by centrifugation. Then, the UV–Vis analysis was taken from the remaining solution to calculate the loaded drug content. For nanoparticles grafted with a polymer chain, the drug loading rate is as follows:

$$\begin{aligned} & {\text{MTX}}\,{\text{drug}}\,{\text{loading}}\,{\text{in}}\,{\text{MSNs - }}g{\text{ - PDMAEMA1}} = \left( {0.{7}/{1}.{14}} \right) \times {1}00 = {61}.{96} \\ & {\text{MTX}}\,{\text{drug}}\,{\text{loading}}\,{\text{in}}\,{\text{MSNs - }}g{\text{ - PDMAEMA5}} = \left( {0.{72}/{1}.{14}} \right) \times {1}00 = {63}.{71} \\ & {\text{DOX}}\,{\text{drug}}\,{\text{loading}}\,{\text{in}}\,{\text{MSNs - }}g{\text{ - PDMAEMA1}} = \left( {0.{3}/0.{5}} \right) \times {1}00 = {6}0 \\ & {\text{DOX}}\,{\text{drug}}\,{\text{loading}}\,{\text{in}}\,{\text{MSNs - }}g{\text{ - PDMAEMA5}} = \left( {0.{32}/0.{5}} \right) \times {1}00 = {64} \\ \end{aligned}$$

### Investigation of drug release at different pH and temperatures

PDMAEMA is a temperature and pH-sensitive polymer; therefore, MTX and DOX release was measured at normal body pH (pH 7.4), tumor pH (pH 5), ambient temperature (25 °C), human body temperature (37 °C), and tumor temperature (41 °C). In this regard, 0.01 g of MSNs-*g*-PDMAEMA with different length of polymer were loaded with MTX in 1 mL of PBS and placed in a dialysis bag. The release of MTX and Dox was investigated at acidic pH (pH 5) and normal body pH (pH 7.4) and at three temperatures 25 ± 1 °C (ambient temperature to store materials in the environment), 37 ± 1 °C (normal body temperature), and 41 ± 1 °C (tumor temperature).

#### Release diagrams of MTX and DOX in MSNs-*g*-PDMAEMA1

**Figure 5:**
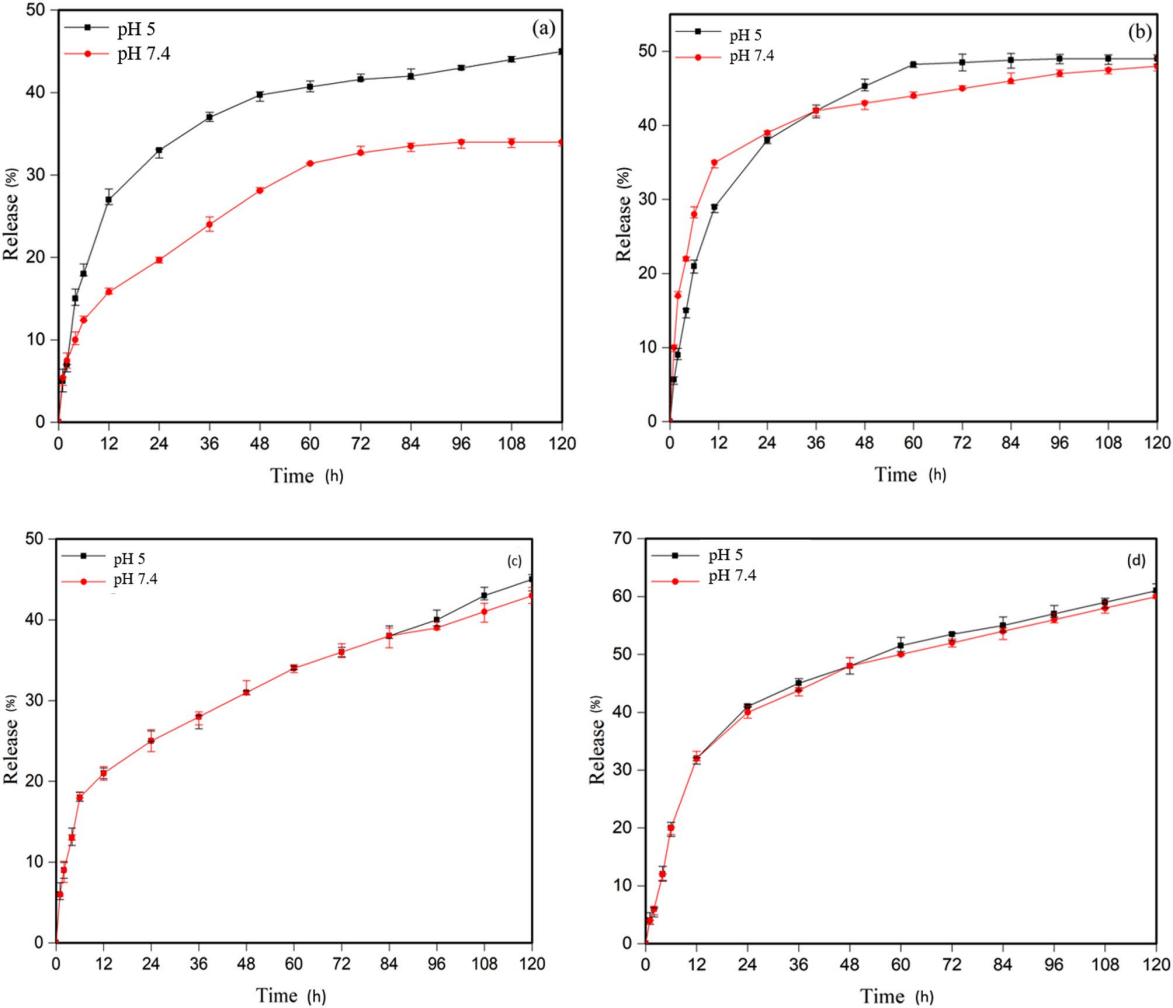
In vitro release profile of (**a**) MTX at T = 25 °C, (**b**) MTX at T = 37 °C, (**c**) DOX at T = 25 °C, and (**d**) DOX at T = 37 °C.

All MTX and DOX drug release analyses were repeated three times to ensure accuracy. The diagrams illustrate the MTX and DOX drug release profiles of MSNs-*g*-PDMAEMA1 are presented in Fig. 5a, b, respectively. The MTX release at both the normal and tumor tissue pH levels was about 40%, while the DOX release was around 60%. This can be attributed to the biphasic nature of the polymer at 25 °C. In Fig. 5c, d, the release rate of MTX at normal body temperature is approximately 50% at both normal and acidic pH, while the release rate of DOX is approximately 58% at both acidic and normal pH at 37 °C. The drug concentration rapidly reaches its peak level within about 12–20 h, followed by a sharp decline. This phenomenon can be attributed to the insufficient coverage of silica pores by the polymer chains that allows for the easy and early release of the drugs molecules.

#### Release diagrams of MTX and DOX in MSNs-*g*-PDMAEMA5

**Figure 6:**
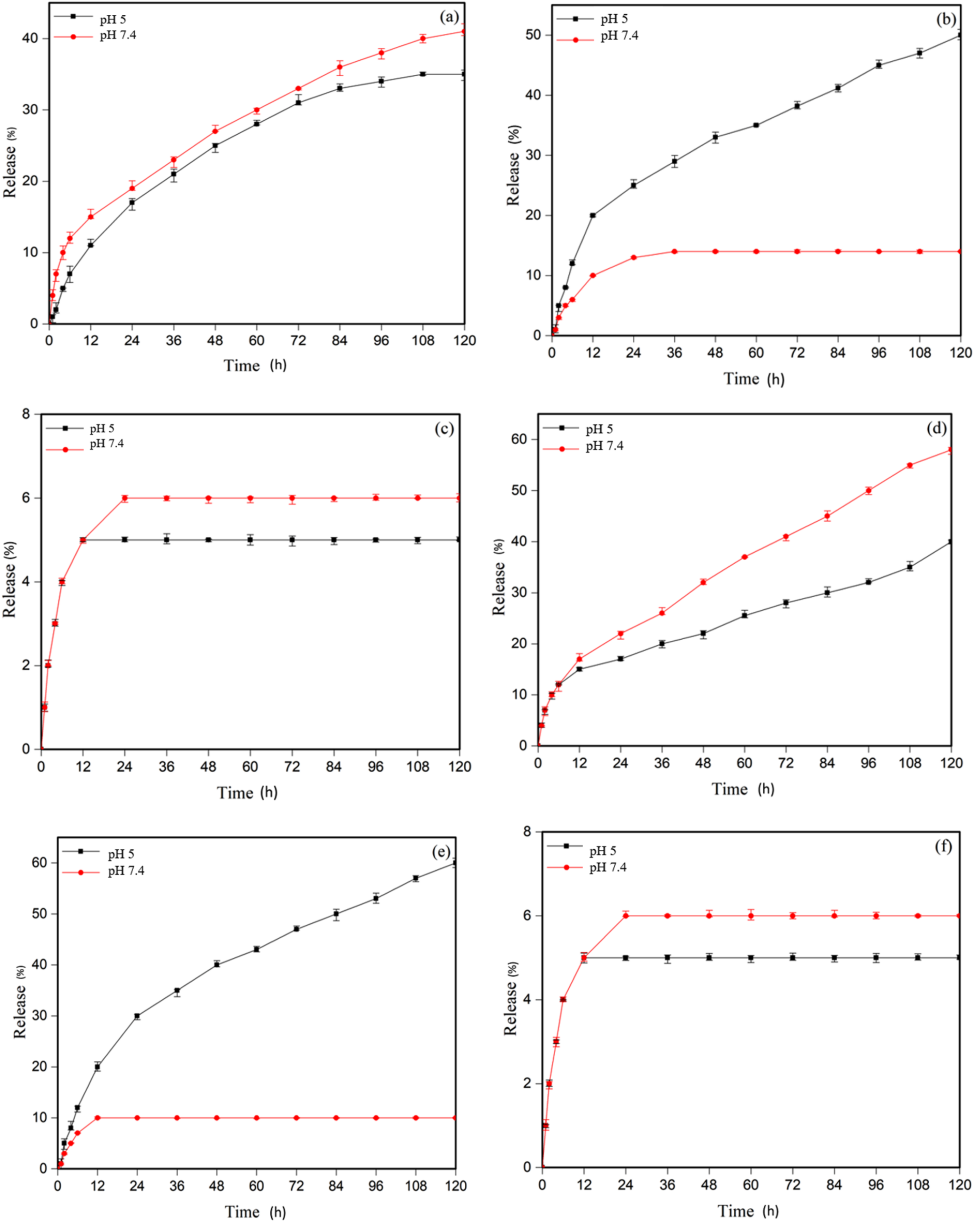
In vitro release profile of (**a**) MTX at T = 25 °C, (**b**) MTX at T = 37 °C, (**c**) MTX at 41 °C, (**d**) DOX at T = 25 °C, (**e**) DOX at T = 37 °C, and (**f**) DOX at T = 41 °C.

The MTX and DOX release diagrams of the grafted nanoparticles in MSNs-*g*-PDMAEMA5 are as follows. As shown in Fig. 6a, b, the drug is still released at 25 °C due to the biphasic polymer at the normal pH, and the release is about 10% lower than the release state at the acidic pH of the tumor. This release is about 15% for DOX. Figure 6c, d relates to release at normal body temperature. The controlled release can be seen at normal body temperature because the polymer is less biphasic, and the chain length is high. At normal body pH, only 12% release was observed, which is about 50% for MTX at acidic pH of the tumor. For DOX at normal body pH, it is 10% and 60% in an acidic environment. So, this system can be considered as a successful system in this regard. Figure 6e, f also correspond to the release at tumor temperature, and due to the single phase of the polymer and low solubility of the drug at this temperature, release of the drug at both pH tumor acidity and normal pH of healthy tissue are below 10%.

MSNs-*g*-PDMAEMA5 exhibits a lower drug release rate in comparison to MSNs-*g*-PDMAEMA1. When MSNs-*g*-PDMAEMA5 is exposed to 37 °C and pH 5 for 12 h, about 20% of MTX was released; while, the release content was around 35% for MSNs-*g*-PDMAEMA1. The release rate of DOX from MSNs-*g*-PDMAEMA5 was significantly slower than that of MSNs-*g*-PDMAEMA1 after 12 h at 37 °C and pH 5. The higher length of the polymer block in MSNs-*g*-PDMAEMA5, impedes the rapid release of drugs in the circulatory system. The gradual release pattern was observed for MSNs-*g*-PDMAEMA5 during the initial time period (0–24 h) that renders it a highly efficient system for delivering cancer drugs. The low loadings of MTX in MCM-41 nanoparticles can be related to the low volume and diameter of the nanoparticles. Due to the low diameter of the cavities, penetration into the depths of the cavities is not possible for the drug. Also, low drug release can be related to the same diameter of cavities. After drug release from larger pores, drug release from smaller pores becomes more complex, and the drug remains in the smaller pores.

### Cytotoxicity of nanoparticles

**Figure 7:**
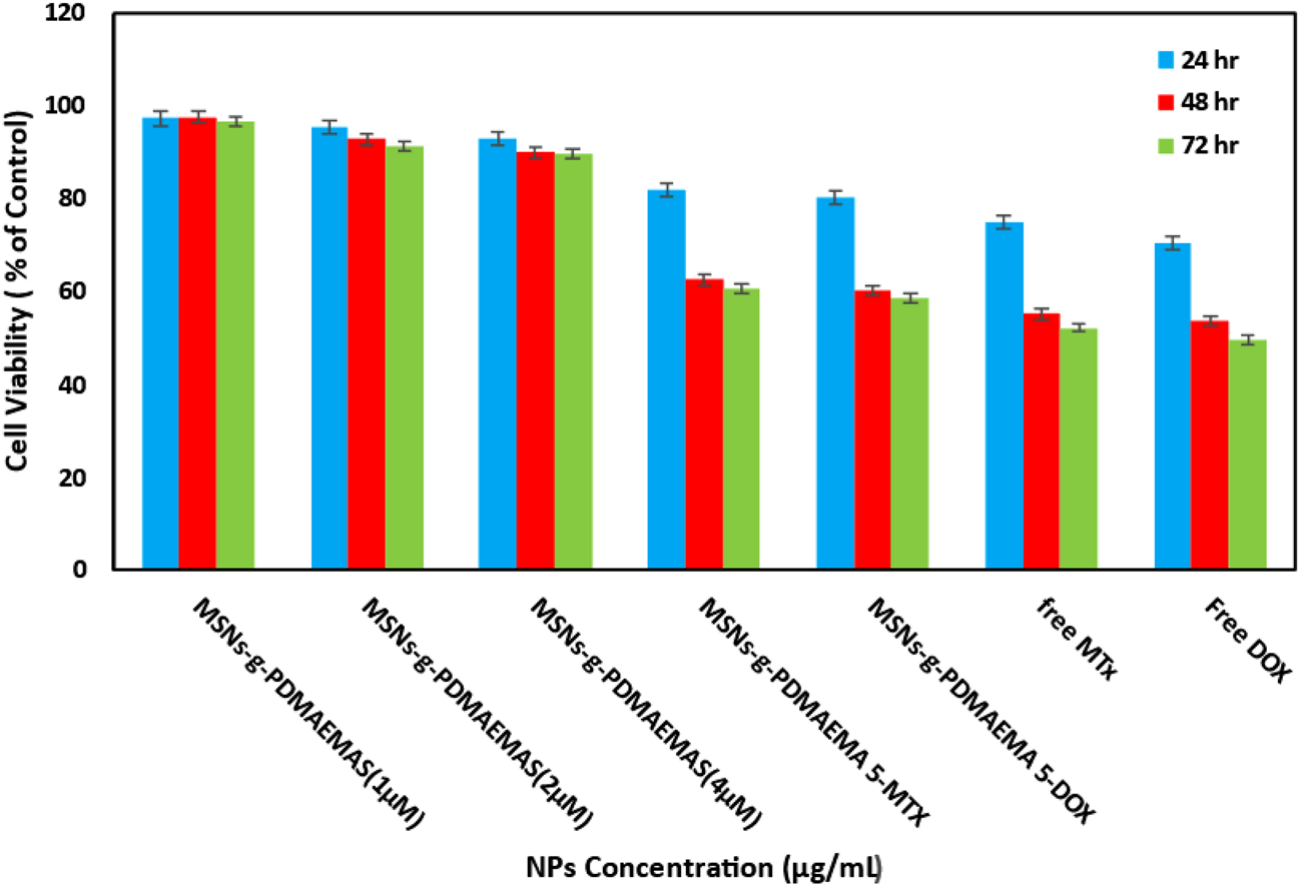
HeLa cancer cells viability after treating with MSNs-*g*-PDMAEMA5 at different concentrations (1–4 μg/mL), MSNs-MTX, MSNs-DOX, and also free MTX and DOX.

Cytotoxicity assays were performed with the HeLa cell line. The cells were treated with MSNs-*g*-PDMAEMA5 with different concentrations and MSNs-*g*-PDMAEMA5-MTX and MSNs-*g*-PDMAEMA5-DOX for 72 h, and then the cell viabilities were recorded. These results are show in Fig. 7. The cell viability was still higher than 90% at different concentrations of the blank carrier after 72 h. This result asserts that the blank carriers have no toxicity for the cells. MSNs-*g*-PDMAEMA5-MTX (5 μM) and MSNs-*g*-PDMAEMA5-DOX (4 μM) were used according to IC50 that killed around 40% of cells after 72 h, showing appropriate release of the drug.

## Conclusions

MCM-41 nanoparticles were functionalized with APTES and 2-bromoisobutyryl bromide to introduce bromine groups. Subsequently, PDMAEMA was grafted onto the MSNs with varying chain lengths. TGA confirmed successful modification steps, with weight reductions of approximately 7% for MCM-41, 14% for MSNs-NH2, 3% for MSNs-Br, and 3% and 12% for MSNs-*g*-PDMAEMA1 and MSNs-*g*-PDMAEMA5, respectively, indicating grafting with two different polymer chains. High molecular weight PDMAEMA chains provided protection for the drug at normal pH and temperature (pH 7.4 and T = 37 °C), while protonation in the tumor environment (pH 5 and T = 37 °C) facilitated drug release. The stretched chain conformation and repulsion between the drug molecules and protonated PDMAEMA chains enhanced the release kinetics. In vitro analyses confirmed the effectiveness of the system in enhancing drug solubility. HeLa cells were used in MTT assays, with MSNs-*g*-PDMAEMA5-DOX and MSNs-*g*-PDMAEMA5-MTX resulting in approximately 40% cell death, indicating successful nanoparticle penetration. The system demonstrated high efficiency in cancer drug delivery, with easy tumor penetration due to proper size and surface charge. The introduction of polymer chains led to a reduction in pore volume, and the modified silica nanoparticles still exhibited a suitable capacity for loading various cancer drugs. The system effectively killed 40% of HeLa cells when loaded with two model drugs, demonstrating excellent controlled release specifically targeting tumor cells. Furthermore, even the blank samples of MSN-g-PDMAEMA5 at three different concentrations resulted in less than 10% cell death in HeLa cells, indicating the high biocompatibility of this system. This robust system can be a candidate for cancer therapy in the future.

### Supplementary Information

Supplementary Figures.

## Data Availability

The datasets generated and/or analyzed during the current study are not publicly available due to confidential agreement with the research collaborators but are available from the corresponding author on reasonable request.
