# Supplementary material for: Dual pH- and temperature-responsive poly(dimethylaminoethyl methacrylate)-coated mesoporous silica nanoparticles as a smart drug delivery system
Source: Sci Rep. 2023 Nov 18;13:20194. doi: 10.1038/s41598-023-47026-7 (PMC10657431; doi:10.1038/s41598-023-47026-7)
Supplement: Supplementary file 1 — Supplementary Figures. [file 41598_2023_47026_MOESM1_ESM.docx]

**Supporting information**

Dual pH- and Temperature-Responsive Poly(dimethylaminoethyl methacrylate)-Coated Mesoporous Silica Nanoparticles as a Smart Drug Delivery System

Sina Ramezanian^1,2^, Jafarsadegh Moghaddas^1,2, *^, Hossein Roghani-Mamaqani^3,4^, Azim Rezamand^5,6^

1. Chemical Engineering Faculty, Sahand University of Technology, P. O. Box 51335-1996, Tabriz, Iran

2. Transport Phenomena Research Center, Chemical Engineering Faculty, Sahand University of Technology, P.O. Box 51335/1996, Tabriz, Iran

3. Faculty of Polymer Engineering, Sahand University of Technology, P.O. Box 51335-1996, Tabriz, Iran

4. Institute of Polymeric Materials, Sahand University of Technology, P.O. Box 51335-1996, Tabriz, Iran

5. Pediatric Health Research Center, Tabriz University of Medical Sciences, Tabriz, Iran

6. Department of Pediatrics, Faculty of Medicine, Tabriz University of Medical Sciences, Tabriz, Iran

Author to whom all correspondence should be addressed. E-mail: [jafar.moghaddas@sut.ac.ir](mailto:jafar.moghaddas@sut.ac.ir) (J. Moghaddas), Tel: (+98-41-33459155).


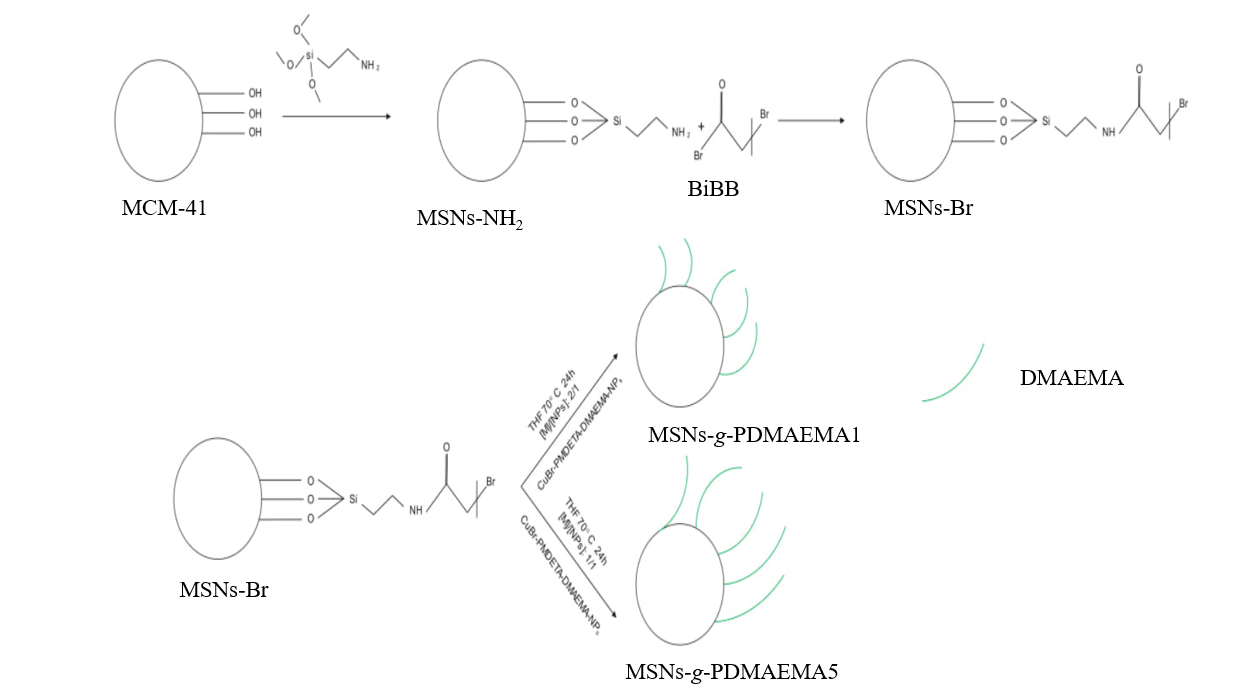


**Figure S1: Synthesis of MSNs-*g*-PDMAEMA**


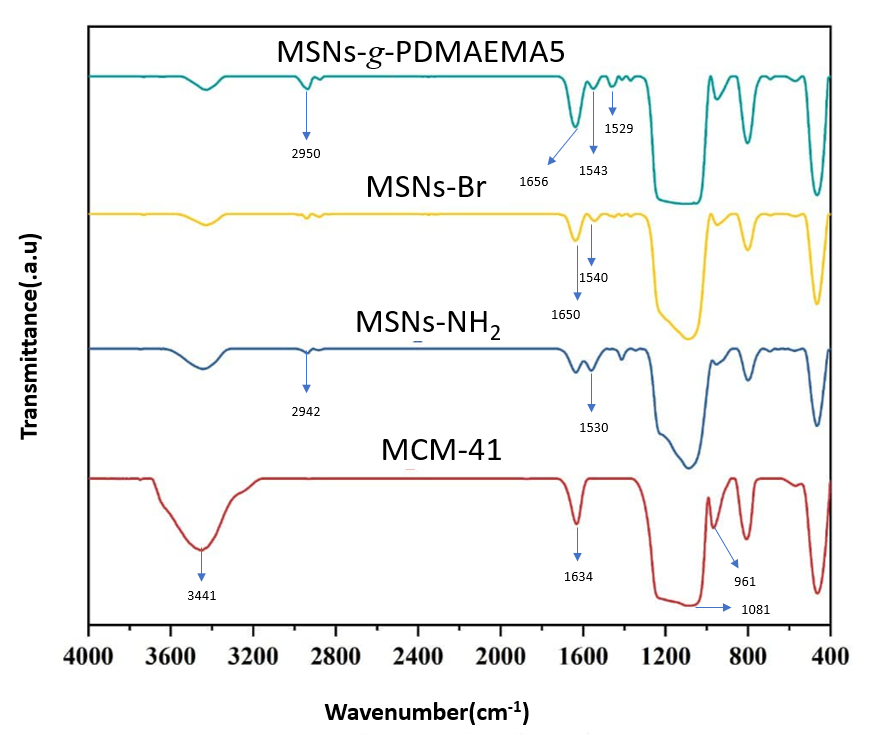


**Figure S2. FT-IR spectra of (a) MCM-41, (b) MSNs-NH_2_, (c) MSNs-Br, and (d) MSNs-*g*-PDMAEMA5**


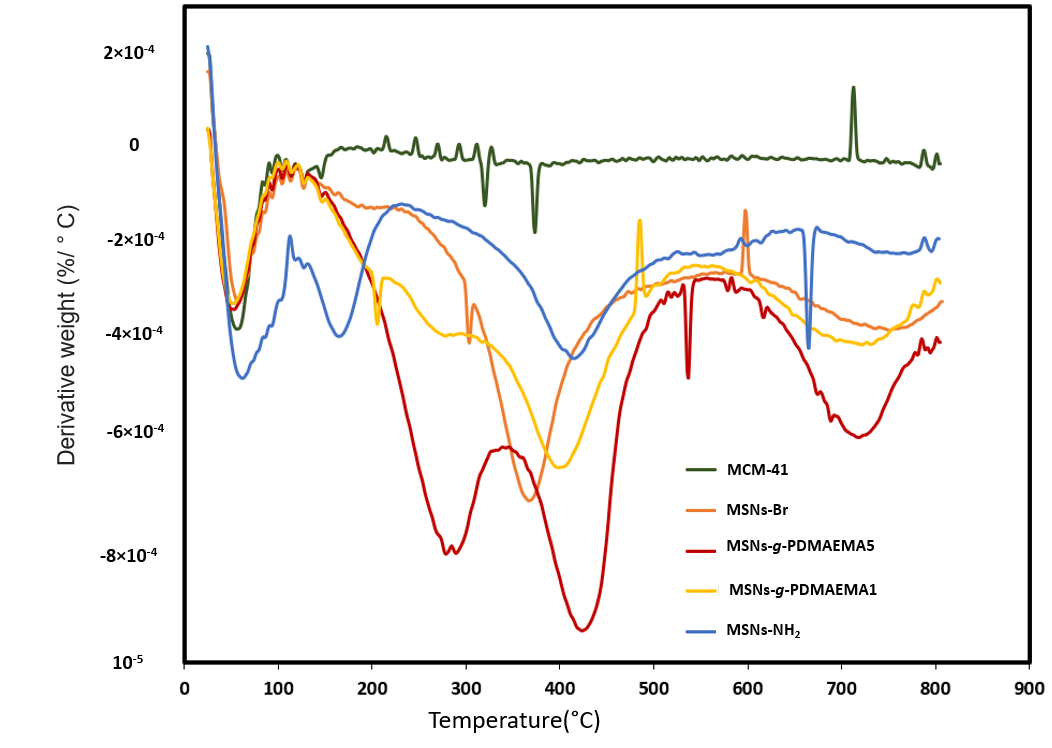


**Figure S3. DTG results for the pure and functionalized MSNs**


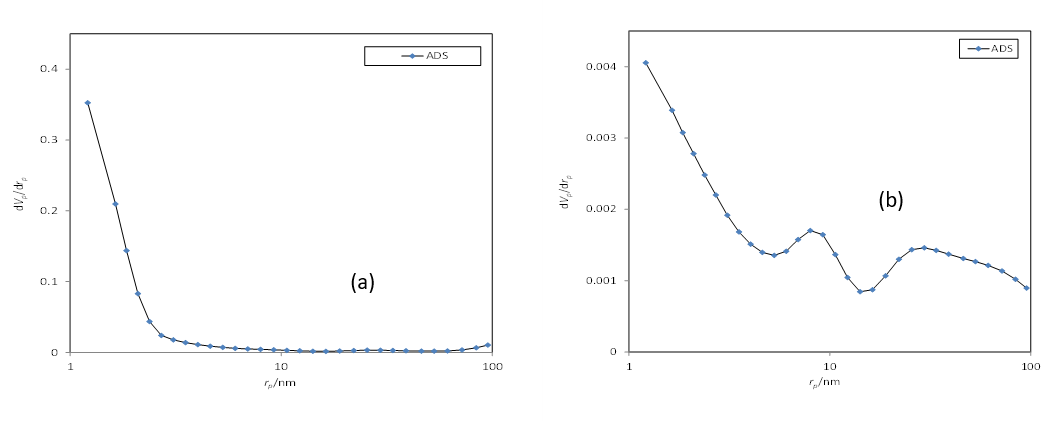

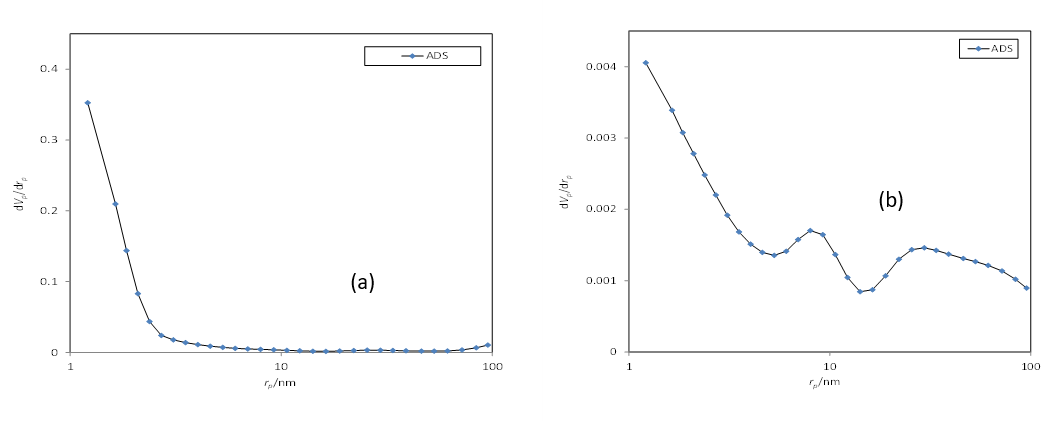


**Figure S4. Pore size distribution diagram related to BJH method for (a) MCM-41 and (b) MSNs-*g*-PDMAEMA5**


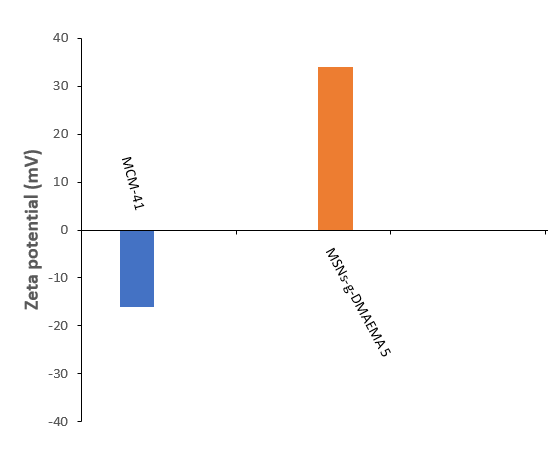


**Figure S5. Zeta potential of MCM-41 and MSNs-*g*-DMAEMA5**
